# The association between ambient UVB dose and ANCA-associated vasculitis relapse and onset

**DOI:** 10.1186/s13075-022-02834-6

**Published:** 2022-06-18

**Authors:** Jennifer Scott, Enock Havyarimana, Albert Navarro-Gallinad, Arthur White, Jason Wyse, Jos van Geffen, Michiel van Weele, Antonia Buettner, Tamara Wanigasekera, Cathal Walsh, Louis Aslett, John D. Kelleher, Julie Power, James Ng, Declan O’Sullivan, Lucy Hederman, Neil Basu, Mark A. Little, Lina Zgaga, on behalf of the RKD and UKIVAS groups, Mark Little, Peter Lavin, Catherine Wall, George Mellotte, Jennifer Scott, Ted Fitzgerald, Hannah O’Keefe, Rachel Dilworth, Pamela O’Neill, Vicki Carr, Niall Conlon, Brenda Griffin, Donal Sexton, Caroline Kosgei, Yvonne O’Meara, Eoghan White, Stephen Mahony, Eamonn Molloy, John Holian, Matt Griffin, David Lappin, Conor Judge, Sarah Cormican, Blathnaid O’Connell, Michelle Clince, Liam Casserly, Michael Clarkson, Michelle O’Shaughnessy, Alyssa Verrelli, Sinead Stoeman, Fergus Daly, Laura Slattery, Aisling Murphy, Declan De Freitas, Peter Conlon, Mark Denton, Carol Treanor, Colm Magee, Conall O. Seaghdha, Paul O’Hara, Susan McGrath, Brona Moloney, Dean Moore, Dearbhla Kelly, Mary McCarthy, Tamara Wanigasekera, Ayanfeoluwa Obilana, Claire Kennedy, Dervla Connaughton, Mark Canney, Limy Wong, Sarah Moran

**Affiliations:** 1 Trinity Health Kidney Centre, Trinity College Dublin, The University of Dublin, Trinity Translational Medicine Institute, St. James’s Street, Dublin 8, Ireland; 2 Institute of Infection, Immunity and Inflammation, College of Medical, Veterinary and Life Sciences, University of Glasgow, Glasgow, UK; 3 ADAPT Centre for Digital Content, Trinity College Dublin, Dublin, Ireland; 4 Department of Statistics, Trinity College Dublin, The University of Dublin, Dublin, Ireland; 5 Royal Netherlands Meteorological Institute (KNMI), De Bilt, The Netherlands; 6 Department of Mathematics and Statistics, University of Limerick, Limerick, Ireland; 7 Department of Mathematical Science, University of Durham, Durham, UK; 8 School of Computer Science, Technological University Dublin, Dublin, Ireland; 9 Vasculitis Ireland Awareness, Galway, Ireland; 10 Department of Public Health and Primary Care, Trinity College Dublin, The University of Dublin, Dublin, Ireland

**Keywords:** ANCA-associated vasculitis, Ultraviolet B (UVB) radiation, Vitamin D, Environment, Geoepidemiology

## Abstract

**Background:**

The aetiology of ANCA-associated vasculitis (AAV) and triggers of relapse are poorly understood. Vitamin D (vitD) is an important immunomodulator, potentially responsible for the observed latitudinal differences between granulomatous and non-granulomatous AAV phenotypes. A narrow ultraviolet B spectrum induces vitD synthesis (vitD-UVB) via the skin. We hypothesised that prolonged periods of low ambient UVB (and by extension vitD deficiency) are associated with the granulomatous form of the disease and an increased risk of AAV relapse.

**Methods:**

Patients with AAV recruited to the Irish Rare Kidney Disease (RKD) (*n* = 439) and UKIVAS (*n* = 1961) registries were studied. Exposure variables comprised latitude and measures of ambient vitD-UVB, including cumulative weighted UVB dose (CW-D-UVB), a well-validated vitD proxy. An *n*-of-1 study design was used to examine the relapse risk using only the RKD dataset. Multi-level models and logistic regression were used to examine the effect of predictors on AAV relapse risk, phenotype and serotype.

**Results:**

Residential latitude was positively correlated (OR 1.41, 95% CI 1.14–1.74, *p* = 0.002) and average vitD-UVB negatively correlated (0.82, 0.70–0.99, *p* = 0.04) with relapse risk, with a stronger effect when restricting to winter measurements (0.71, 0.57–0.89, *p* = 0.002). However, these associations were not restricted to granulomatous phenotypes. We observed no clear relationship between latitude, vitD-UVB or CW-D-UVB and AAV phenotype or serotype.

**Conclusion:**

Our findings suggest that low winter ambient UVB and prolonged vitD status contribute to AAV relapse risk across all phenotypes. However, the development of a granulomatous phenotype does not appear to be directly vitD-mediated. Further research is needed to determine whether sufficient vitD status would reduce relapse propensity in AAV.

**Supplementary Information:**

The online version contains supplementary material available at 10.1186/s13075-022-02834-6.

## Introduction

Anti-neutrophil cytoplasm antibody (ANCA)-associated vasculitis (AAV) is a rare multi-system autoimmune disorder, with an annual incidence of 13–20 cases per million [1].

It manifests as one of three main phenotypes: granulomatosis with polyangiitis (GPA), microscopic polyangiitis (MPA) or eosinophilic granulomatosis with polyangiitis (EGPA). Characteristic autoantibodies directed against myeloperoxidase (MPO) or proteinase-3 (PR3) molecules determine the ANCA serotype. It follows a relapsing and remitting course, resulting in cumulative tissue damage. While the precise triggers of AAV onset and relapse are unknown, evidence supports a complex interaction of genetic susceptibility loci [2], epigenetics [3] and environmental factors [4].

A potential effect of ultraviolet (UV) radiation on disease phenotype and disease activity has been proposed, as marked variations in GPA:MPA incidence ratios occur with latitude [5–8]. Increasing latitude (and hence decreasing UV) [8] is associated with an increased incidence of GPA and EGPA subtypes [5, 8] and of anti-PR3 positivity [9]. The magnitude of association may be strongest for winter UV levels [8], because it is these that particularly limit cutaneous vitamin D (vitD) synthesis [10] and the strongest latitudinal gradient in UV is observed in winter [11]. A similar inverse correlation between UV radiation exposure and incidence is observed in other autoimmune diseases (ADs), for example, multiple sclerosis [12, 13] and type 1 diabetes [12]. In contrast, for the MPA phenotype, an increasing incidence with *decreasing* latitude was observed [5, 6], although Gatenby et al. could not replicate this relationship (nor did they identify an association with UV radiation) [8].

vitD has immunomodulatory effects, including suppression of Th1 and Th17 cells and upregulation of Treg cells, resulting in the reduction of inflammatory cytokines and a more tolerogenic environment [4, 14–16]. Th1, Th17 and Treg cells are crucial to granuloma formation, characteristically seen in GPA and EGPA, but not in MPA, where Th2 cells and antibody-mediated mechanisms predominate [17]. Gatenby et al. postulated that this vitD-mediated UV effect on granulomas was responsible for the strong inverse relationship between GPA (but not MPA) incidence and UV radiation, but this hypothesis has not been tested.

Several small studies demonstrate that serum 25-hydroxyvitamin D (25OHD) levels are lower in AAV patients than in healthy controls [18–20]. Low 25OHD levels have been associated with increased disease activity in other ADs [21–23], with suggestive data in AAV [23]. To our knowledge, there are no large studies examining the influence of UV radiation or vitD on AAV disease activity, as distinct from ANCA phenotype or serotype. Ambient UVB dose at wavelengths that induce cutaneous vitD_3_ synthesis (vitD-UVB) is the key determinant of an individual’s vitD status. Repeated 25OHD assessment is costly and not routinely performed. vitD levels also drop with inflammation limiting the value of 25OHD measurement at relapse. Therefore, we chose to use the cumulative-weighted ambient UVB dose (CW-D-UVB)—a well-validated predictor of an individual’s vitD status [24–27]—as a surrogate.

The aim of this study is, firstly, to examine the association between measures of ambient UV radiation, UVB-predicted vitD status (CW-D-UVB) and AAV *relapse*, using a novel *n*-of-1 case-control design. Secondly, we aim to explore the relationship between these variables and ANCA *phenotype and serotype at diagnosis*. We hypothesise that prolonged periods of low ambient UVB (and by extension vitD deficiency) are associated with an increased risk of AAV relapse in GPA/EGPA, but not MPA, and an increased risk of a GPA/EGPA phenotype at diagnosis.

## Methods

### Study participants

*UKIVAS cohort 1* comprised patients from the United Kingdom and Ireland Vasculitis Rare Disease Group (UKIVAS) Registry (https://ukivas.ndorms.ox.ac.uk/), established in 2009. Patients were included in this study if they had a diagnosis of definite AAV with documented diagnosis date and location, defined by relevant clinical features of GPA, MPA or EGPA [28], with either positive anti-MPO or PR3 serology [29] and/or diagnostic histopathology. Patients with secondary vasculitis or dual anti-glomerular basement membrane disease were excluded [30].

*RKD cohort 2* comprised non-overlapping patients from the national Irish Rare Kidney Disease (RKD) Registry and Biobank (https://www.tcd.ie/medicine/thkc/research/rare.php), established in 2012. The same inclusion and exclusion criteria were applied. For the AAV relapse component, participants were also required to have > 1 documented period of remission prior to the commencement of the analysis on 18 August 2020. Participant selection is outlined in Additional file 1: Figs. S1 and S2. The AAV diagnosis study included both cohorts, while the AAV relapse study included RKD only, as granular longitudinal follow-up was not recorded in UKIVAS. Ethical approval for the RKD and UKIVAS components was granted by the Tallaght University Research Ethics Committee (ref 2019-08 List 29 (07)) and the NHS National Research Ethics Service (REC 10/H1102/77), respectively. All participants provided written informed consent.

### Participant location

Additional file 1: Fig. S3 shows the participant locations at diagnosis. We employed a smartphone application for the RKD cohort (patientMpower) [31] to capture participants’ location by GPS [32]. If unavailable at the time of assessment, the electoral division of the participant’s residence or their hospital was used (ranked in preference order). Data linkage was performed using a Resource Description Framework (RDF) semantic web model [33]. Participants’ residential postcodes informed the location coordinates for the UKIVAS cohort.

### Ultraviolet B (UVB) data source, measures of ambient vitD-UVB and cumulative-weighted UVB dose (CW-D-UVB)

**Fig. 1:**
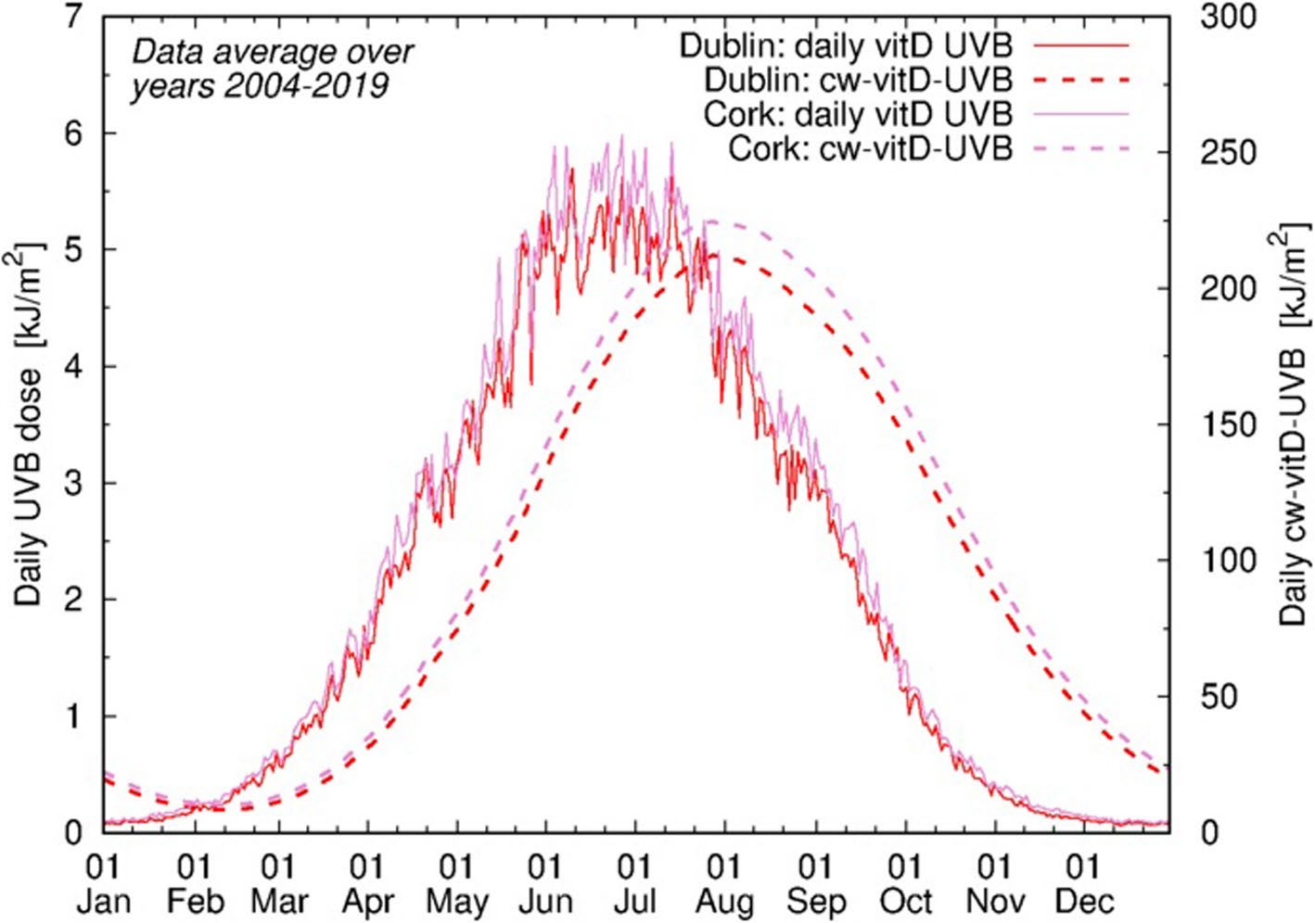
Annual distribution of average daily CW-D-UVB (dashed line) relative to the average daily vitD-UVB (solid line). Values in Dublin (red) and Cork (pink), averaged over 2004–2019, are displayed. The annual peak and nadir of CW-D-UVB were observed in August and February, respectively, lagging behind those of vitD-UVB by 2 months, thus mimicking 25OHD seasonal fluctuations

Daily UVB data at wavelengths specific for vitD production (vitD-UVB) were obtained from the Tropospheric Emission Monitoring Internet Service (TEMIS) database [34, 35]. To generate UVB-predicted vitamin D status, a specific cumulative-weighted UVB dose (CW-D-UVB) was calculated for each participant, determined by high-resolution location and date, based on an algorithm proposed by Kelly et al. [24] and later validated [25–27]. Briefly, a daily vitD-UVB dose over 135 days before a given date was used with a decay function assuming a vitD half-life of 35 days (Fig. 1). Further detail is provided in the supplementary materials.

The average (2004–2019) annual, average winter and preceding winter vitD-UVB and CW-D-UVB were derived for each participant. Winter measures were specifically included given the stronger effect sizes previously noted [8].

### Study design

**Fig. 2:**
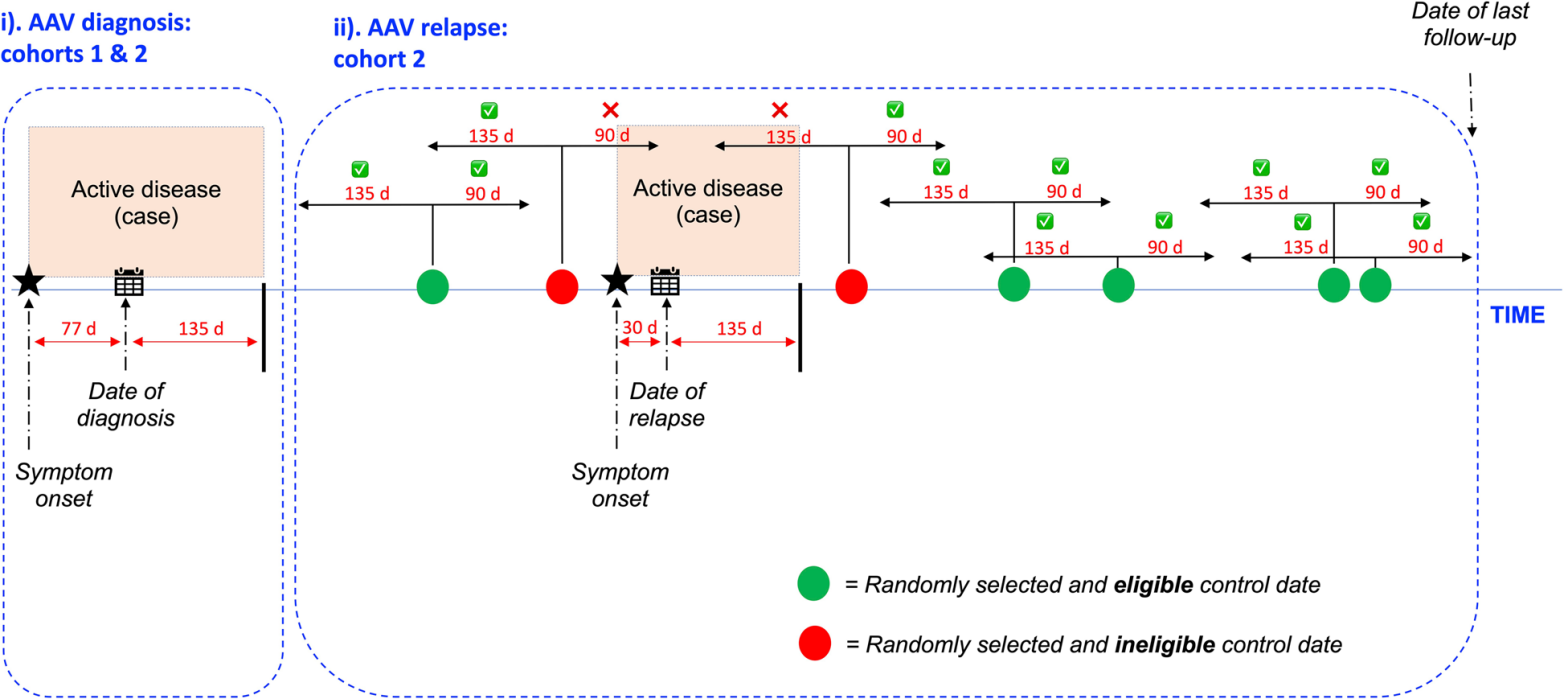
Study design. **i** *AAV diagnosis* (cohorts 1 and 2). **ii** *AAV relapse* (cohort 2). **i** CW-D-UVB at diagnosis was calculated using the participant’s location and date of symptom onset if known, or the date of diagnosis minus 77 days. Seventy-seven days represents the undefined prodromal period [36] informed by the RKD registry analysis (see supplementary material). **ii** In this prospective *n*-of-1 component, each participant was a ‘case’ during period(s) of disease relapse and a ‘control’ during period(s) of remission [37]. The case window started at the date of relapse diagnosis minus 30 days (to account for the diagnostic delay) and ended 135 days later (see Additional file 1: Supplementary Methods). To improve the statistical power, 5 control dates were identified per patient, where possible

The study was split into AAV relapse and diagnosis components, further described in Fig. 2 and supplementary materials. Briefly, relapse was studied using an *n*-of-1 design where each participant acts as their own control, eliminating confounding by time-independent factors such as gender, skin type and occupation and genotype. Case (relapse) and control (remission) windows were defined using an algorithm that considered the effect of residual disease activity and the CW-D-UVB interval (135 days). The diagnosis was studied using the estimated date of symptom onset. Relapse was defined as the return of signs and/or symptoms of active vasculitis (BVAS > 0), with supporting laboratory or histopathological evidence, escalation in immunosuppression and a clinical response to the same. Relapse events were adjudicated by a committee (JS and ML), blinded to the individual’s UV data. Remission was defined as the absence of signs, symptoms and laboratory evidence of vasculitis activity. Follow-up continued until the last documented visit or death.

### Patient and Public Involvement

Patient and Public Involvement is detailed in supplementary materials.

### Statistical analysis

#### AAV relapse

A multi-level model (*lme4* package, *glmer* function) [38] was applied to investigate the association between latitude/measures of ambient vitD-UVB/CW-D-UVB and AAV relapse. Covariates included age, gender, AAV phenotype, ANCA serotype and treatment. A random effect was included to account for repeated measures and the varying relapse risk between individuals [39]. The disease phenotype was included as an interaction term, to study the (hypothesised) effect of exposure variables on disease subtype relapse risk. Sensitivity analysis restricted to (non-) granulomatous phenotypes was also performed. The power calculation is detailed in supplementary materials.

#### AAV diagnosis

Logistic regression was used to examine the relationships between AAV phenotype and serotype (outcome variables), latitude, measures of ambient vitD-UVB and CW-D-UVB (examined separately due to collinearity). Covariates included age, gender and ethnicity. We performed a sensitivity analysis restricted to Whites only, given their more substantial vitD synthesis in the skin.

Odds ratios (ORs) and 95% confidence intervals (CIs) were reported. *p* < 0.05 was considered statistically significant. All statistical analyses were performed using R (version 4.0.4).

## Results

### Participant characteristics

UKIVAS cohort 1 (*N* = 1961, Table 1) and RKD cohort 2 (*N* = 439, Table 2) were similar. An excess of non-White and GPA participants in UKIVAS were the main differences. Induction and maintenance treatment are detailed in Additional file 1: Table S1. The Median follow-up time in RKD was 58.3 months (IQR 32.1–138.1). A total of 343 (78.1%) patients did not relapse; there were 135 relapse events in 96 patients.

**Table 1:** Baseline characteristics of cohort 1 (UKIVAS)

Characteristics	Total	GPA	MPA	EGPA	***p***
***n (%)***	1961	1124 (57.3)	600 (30.6)	237 (12.1)	
**Age (years, median [IQR])**	60 [49, 69]	58 [47, 67]	66 [57, 73]	57 [47, 65]	< 0.001
**Male (%)**	1017 (51.9)	610 (54.3)	280 (46.7)	127 (53.6)	0.009
***Ethnicity (%)***					< 0.001
**White**	1796 (91.6)	1049 (93.3)	534 (89.0)	213 (89.9)	
**Asian**	85 (4.3)	44 (3.9)	34 (5.7)	7 (3.0)	
**Black**	29 (1.5)	4 (0.4)	16 (2.7)	9 (3.8)	
**Mixed**	9 (0.5)	5 (0.4)	3 (0.5)	1 (0.4)	
**Others**	42 (2.1)	22 (2.0)	13 (2.2)	7 (3.0)	
***ANCA subtype (ELISA) (%)***					< 0.001
**PR3**	499 (25.4)	437 (38.9)	53 (8.8)	9 (3.8)	
**MPO**	340 (17.3)	52 (4.6)	240 (40.0)	48 (20.3)	
**ELISA-negative**	63 (3.2)	21 (1.9)	15 (2.5)	27 (11.4)	
**Others**	6 (0.3)	3 (0.3)	2 (0.3)	1 (0.4)	
**No ELISA recorded**	1053 (53.7)	611 (54.4)	290 (48.3)	152 (64.1)	
**Latitude (degrees, median [IQR])**	52.24 [51.50, 53.38]	52.40 [51.54, 53.42]	51.82 [51.46, 52.94]	52.24 [51.44, 53.48]	< 0.001
**Longitude (degrees, median [IQR])**	− 1.20 [− 2.23, − 0.14]	− 1.48 [− 2.36, − 0.20]	− 0.57 [− 2.00, − 0.10]	− 1.38 [− 2.20, − 0.18]	< 0.001
**CW-D-UVB at symptom onset (kJ/m**^**2**^**, median [IQR])**	83.82 [25.30, 168.57]	80.72 [25.03, 167.62]	88.40 [26.50, 167.44]	82.51 [24.50, 172.69]	0.914
***Season of diagnosis (%)***					0.891
**Spring**	490 (25.0)	285 (25.4)	143 (23.8)	62 (26.2)	
**Summer**	527 (26.9)	307 (27.3)	161 (26.8)	59 (24.9)	
**Autumn**	480 (24.5)	274 (24.4)	144 (24.0)	62 (26.2)	
**Winter**	464 (23.7)	258 (23.0)	152 (25.3)	54 (22.8)	

Continuous variables are reported as mean (standard deviation (SD)) or median (interquartile range (IQR)) if not normally distributed and compared using the independent sample *t*-test or the Mann-Whitney *U* test, respectively. Categorical variables are summarised by frequency and percentage (%) and compared using the *χ*^2^ test

Refer to supplementary materials for definitions of ‘date of symptom onset’ and seasons

*ANCA* anti-neutrophil cytoplasmic antibodies, *ELISA* enzyme-linked immunoassay, *PR3* proteinase-3, *MPO* myeloperoxidase, *CW-DUVB* cumulative-weighted UVB dose, *SD* standard deviation, *IQR* inter-quartile range

**Table 2:** Baseline characteristics of cohort 2 (RKD)

Characteristics	Total	GPA	MPA	EGPA	***p***
*n (%)*	439	196 (44.6)	220 (50.1)	23 (5.2)	
Age (years, median [IQR])	59.0 [48.0, 69.0]	54.0 [40.0, 62.3]	65.0 [54.0, 73.0]	57.0 [51.5, 62.5]	< 0.001
Male (%)	253 (57.6)	112 (57.1)	129 (58.6)	12 (52.2)	0.823
Ethnicity (%)					0.353
White	433 (98.6)	194 (99.0)	217 (98.6)	22 (95.7)	
Asian	6 (1.4)	2 (1.0)	3 (1.4)	1 (4.3)	
*ANCA subtype (ELISA) (%)*					< 0.001
PR3	219 (49.9)	170 (86.7)	45 (20.5)	4 (17.4)	
MPO	207 (47.2)	19 (9.7)	172 (78.2)	16 (69.6)	
ELISA-negative	12 (2.7)	6 (3.1)	3 (1.4)	3 (13.0)	
No ELISA recorded	1 (0.2)	1 (0.5)	0 (0.0)	0 (0.0)	
*Organ involvement (%)*					
Musculoskeletal	168 (38.3)	103 (52.6)	53 (24.1)	12 (52.2)	< 0.001
Mucocutaneous	115 (26.2)	71 (36.2)	34 (15.5)	10 (43.5)	< 0.001
Eyes	57 (13.0)	37 (18.9)	18 (8.2)	2 (8.7)	0.004
Lung	229 (52.2)	132 (67.3)	78 (35.5)	19 (82.6)	< 0.001
Neurological	60 (13.7)	27 (13.8)	22 (10.0)	11 (47.8)	< 0.001
Ears, nose, throat	193 (44.0)	147 (75.0)	29 (13.2)	17 (73.9)	< 0.001
Cardiovascular	12 (2.7)	6 (3.1)	6 (2.7)	0 (0.0)	1
Kidney	370 (84.3)	146 (74.5)	212 (96.4)	12 (52.2)	< 0.001
Gastrointestinal	25 (5.7)	13 (6.6)	11 (5.0)	1 (4.3)	0.828
Latitude (degrees, median [IQR])	53.30 [52.67, 53.39]	53.29 [52.67, 53.39]	53.32 [52.76, 53.39]	53.29 [52.86, 53.37]	0.350
Longitude (degrees, median [IQR])	− 6.39 [− 7.77, − 6.30]	− 6.43 [− 8.37, − 6.27]	− 6.48 [− 8.35, − 6.22]	− 6.39 [− 7.77, − 6.30]	0.684
CW-D-UVB at symptom onset (kJ/m^2^, median [IQR])	75.98 [19.12, 161.06]	78.46 [20.29, 166.07]	74.57 [18.94, 153.51]	60.41 [30.27, 115.27]	0.842
*Season of diagnosis (%)*					0.502
Spring	129 (29.4)	60 (30.6)	67 (30.5)	2 (8.7)	
Summer	92 (21.0)	41 (20.9)	44 (20.0)	7 (30.4)	
Autumn	112 (25.5)	48 (24.5)	57 (25.9)	7 (30.4)	
Winter	106 (24.1)	47 (24.0)	52 (23.6)	7 (30.4)	
Follow-up (months, median [IQR])	58.3 [32.1, 138.1]	84.7 [39.5, 183.9]	45.4 [26.0, 83.8]	108.4 [49.8, 160.2]	< 0.001
Death (%)	38 (8.7)	11 (5.6)	25 (11.4)	2 (8.7)	0.112
*Number of patients experiencing n relapse(s) (%)*					
0	343 (78.1)	141 (71.9)	188 (85.5)	14 (60.9)	
1	67 (15.3)	37 (18.9)	26 (11.8)	4 (17.4)	
2	20 (4.6)	12 (6.1)	4 (1.8)	4 (17.4)	
3	8 (1.8)	5 (2.6)	2 (0.9)	1 (4.3)	
4	1 (0.2)	1 (0.5)	0 (0)	0 (0)	

Refer to supplementary materials for the definitions of ‘date of symptom onset’ and seasons. Refer to Additional file 1: Table S1 for the details on the induction and maintenance treatment. Continuous variables are reported as mean (standard deviation (SD)) or median (interquartile range (IQR)) if not normally distributed and compared using the independent sample *t*-test or the Mann-Whitney *U* test, respectively. Categorical variables are summarised by frequency and percentage (%) and compared using the *χ*^2^ test

*ANCA* anti-neutrophil cytoplasmic antibodies, *ELISA* enzyme-linked immunoassay, *PR3* proteinase-3, *MPO* myeloperoxidase, *CW-DUVB* cumulative-weighted UVB dose, *SD* standard deviation, *IQR* inter-quartile range

### UVB patterns

The annual peak and nadir of CW-D-UVB were observed in August and February, respectively, lagging behind those of vitD-UVB by 2 months thus mimicking 25OHD seasonal fluctuations (Fig. 1 and Additional file 1: Fig. S4). The *daily average* vitD-UVB and CW-D-UVB were strongly correlated with latitude (Additional file 1: Fig. S5a-b), as expected. However, latitude alone was weakly correlated with vitD-UVB or CW-vitD-UVB *on a particular day (e.g. diagnosis)*, because the impact of the season is much greater than latitude. This correlation remained weak even when stratified by season due to large variation in daily UVB (Additional file 1: Fig. S5c-g), thus strengthening the rationale for using CW-D-UVB as vitD proxy, over latitude or season alone.

### Impact of latitude, measures of ambient vitD-UVB and cumulative-weighted UVB (CW-D-UVB) on AAV relapse

**Fig. 3:**
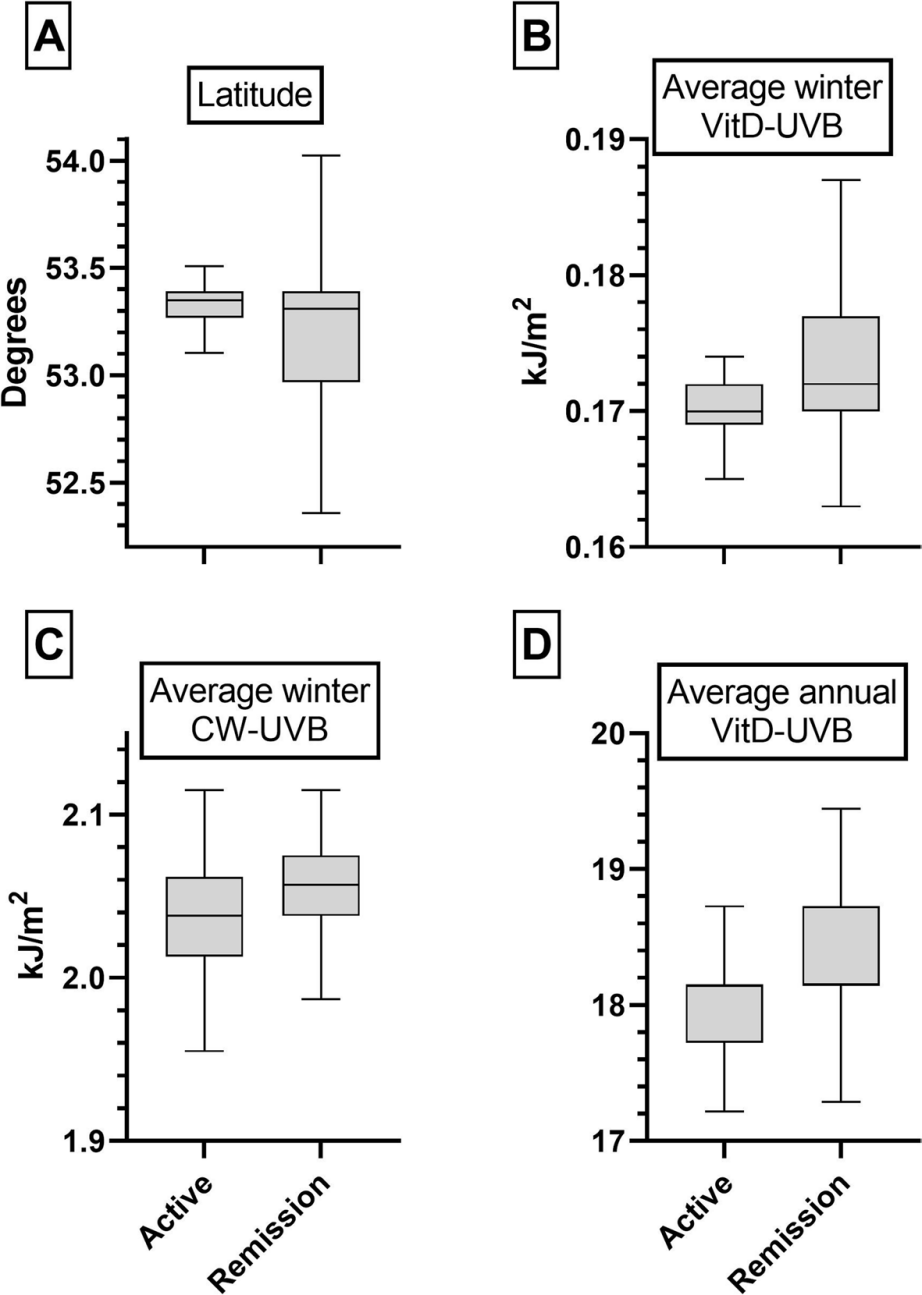
*AAV relapse*. **a** Latitude (degrees), **b** average winter vitD-UVB (kJ/m^2^), **c** average winter CW-D-UVB (kJ/m^2^) and **d** average annual vitD-UVB (kJ/m^2^) stratified by disease activity (active vs. remission) in the entire cohort 2

**Fig. 4:**
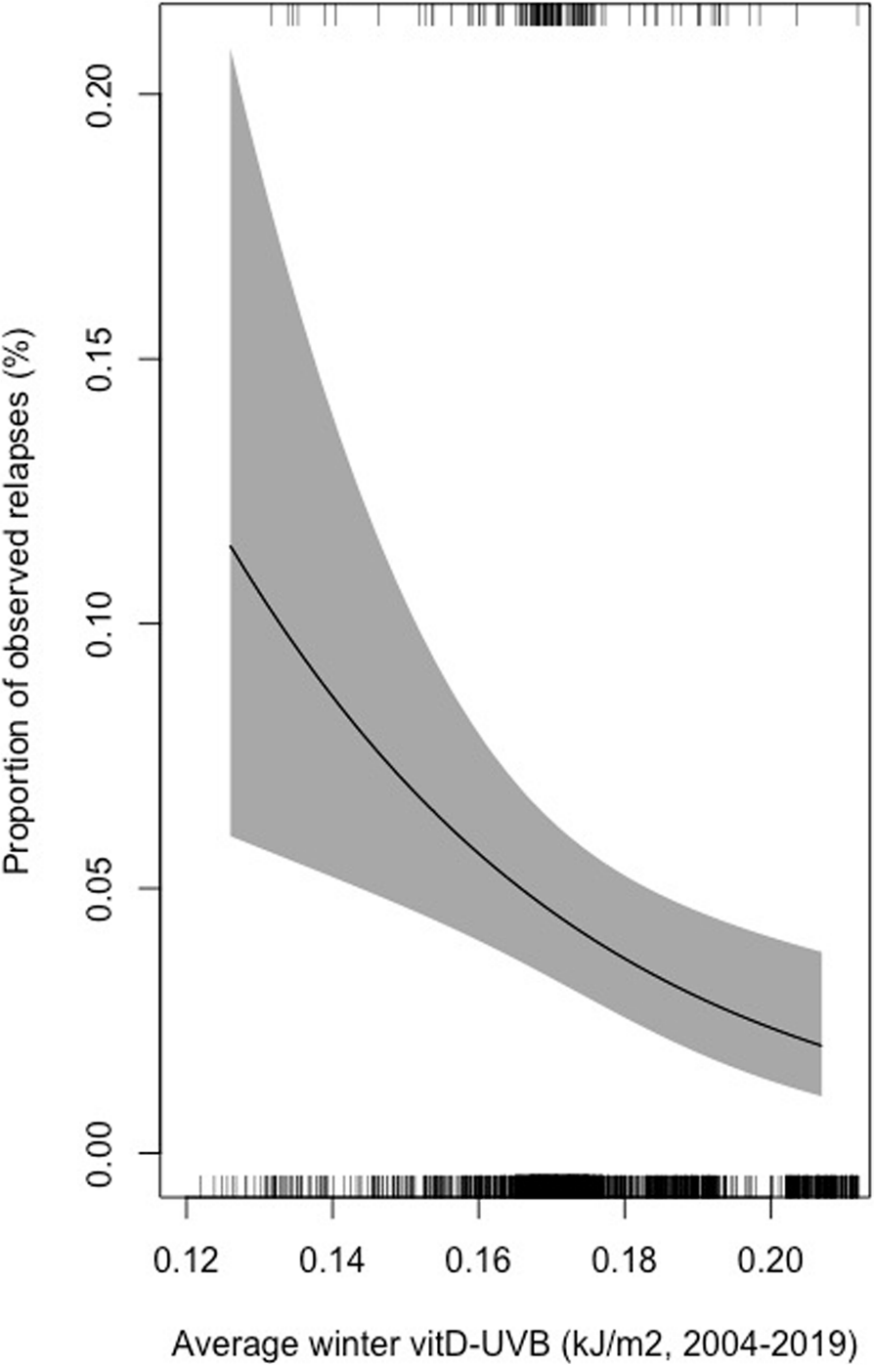
Effects plot demonstrating the marginal effect of average winter vitD-UVB (kJ/m2) on relapse risk. This is a graphical representation of the multi-level model reported in Table 2 (model 2), controlling for age at diagnosis, gender, AAV phenotype, ANCA serotype and treatment status. The average value for continuous covariates and the baseline value for categorical covariates are depicted. Ticks at the top and bottom of the graph refer to the relapse and remission events, respectively. The *predictorEffect* function from the *effects* R package [40, 41] was adapted to create this graphic

Using multi-level logistic regression analysis, latitude was associated with AAV relapse risk (OR 1.41, 95% CI 1.14–1.74, Table 3 and Fig. 3a). There was also a significant inverse relationship between relapse risk and average winter vitD-UVB (0.71, 0.57–0.89, Figs. 3b and 4) and annual vitD-UVB (0.82, 0.70–0.99, Fig. 3d), with a slightly larger effect size in MPA versus not-MPA (Additional file 1: Table S2). However, no relationship was observed with *preceding winter* vitD-UVB (0.90, 0.74–1.10, Table 3 and Additional file 1: Fig. S6b). Similarly, we noted a significant inverse relationship between relapse risk and both average (0.82, 0.70–0.99, Fig. 3c) and preceding (0.81, 0.66–0.99, Additional file 1: Fig. S6c) winter CW-D-UVB. The median CW-D-UVB at symptom onset was not significantly associated with relapse, overall or when stratified by AAV phenotype and ANCA serotype (1.06, 0.88–1.28, Additional file 1: Fig. S6a). The addition of an interaction term between AAV phenotype and any exposure variable did not alter the findings. Additional independent risk factors for relapse included GPA/EGPA subtype (1.78, 1.03–3.05), younger age (0.75, 0.61–0.92) and being off immunosuppression therapy (2.65, 1.70–4.14) or on glucocorticoid monotherapy (1.85, 1.02–3.35, Additional file 1: Table S3).

**Table 3:** Multi-level models investigating the factors associated with AAV relapse risk

	Latitude	Average winter (2004–2019)		Average annual vitD-UVB	CW-D-UVB at symptom onset	Preceding winter	
		vitD-UVB	CW-D-UVB			vitD-UVB	CW-D-UVB
	Model 1	Model 2	Model 3	Model 4	Model 5	Model 6	Model 7
**Random effects (variance (SD))**							
Patient ID	0.55 (0.75)	0.56 (0.75)	0.57 (0.76)	0.60 (0.78)	0.64 (0.80)	0.59 (0.77)	0.54 (0.74)
**Fixed effects (OR (95% CI,** ***p*))**							
Latitude (degrees)	1.41 (1.14–1.74, **0.002)**	–	–	–	–	–	–
Average winter vitDUVB (kJ/m^2^)	–	0.71 (0.57–0.89, **0.002**)	–	–	–	0.90 (0.74–1.10, 0.31)	–
Average winter CWD-UVB (kJ/m^2^)	–	–	0.74 (0.60–0.91, **0.005**)	–	–	–	0.81 (0.66–0.99, **0.04)**
Average annual vitDUVB (kJ/m^2^)	–	–	–	0.82 (0.70–0.99, **0.04**)	–	–	–
CW-D-UVB at symptom onset (kJ/m^2^)	–	–	–	–	1.06 (0.88–1.28, 0.52)	–	–
Not MPA (ref: MPA)	1.78 (1.03–3.05, **0.04)**	1.72 (1.00–2.96, **0.05**)	1.75 (1.01–3.01, **0.05**)	1.74 (1.01–3.02, **0.048**)	1.79 (1.02–3.13, **0.04**)	1.78 (1.03–3.10, **0.04**)	1.78 (1.03–3.07, **0.04**)
Age at diagnosis (years)	0.75 (0.61–0.92, **0.006**)	0.74 (0.60–0.90, **0.004**)	0.74 (0.60–0.91, **0.004**)	0.73 (0.60–0.90, **0.004**)	0.73 (0.59–0.90, **0.003**)	0.74 (0.60–0.90, **0.004**)	0.74 (0.60–0.90, **0.004**)
Gender (male)	0.93 (0.61–1.41, 0.73)	0.92 (0.61–1.39, 0.70)	0.91 (0.60–1.38, 0.67)	0.91 (0.60–0.90, 0.64)	0.90 (0.60–1.38, 0.65)	0.91 (0.60–1.37, 0.65)	0.91 (0.60–1.37, 0.65)
Not MPO-ANCA (ref: MPO-ANCA)	1.10 (0.64–1.86, 0.74)	1.10 (0.65–1.88, 0.72)	1.10 (0.64–1.87, 0.74)	1.10 (0.64–1.89, 0.73)	1.08 (0.63–1.87, 0.77)	1.08 (0.63–1.86, 0.77)	1.08 (0.63–1.83, 0.79)
Off treatment (ref: On treatment)	2.65 (1.70–4.14, **< 0.001**)	2.65 (1.70–4.13, **< 0.001)**	2.66 (1.70–4.16, **< 0.001)**	2.66 (1.70–4.17, **< 0.001**)	2.62 (1.68–4.11, **< 0.001**)	2.65 (1.70–4.14, **< 0.001)**	2.64 (1.70–4.11, **< 0.001)**
Number of individuals	439	439	439	439	439	439	439
Number of observations	2080	2080	2080	2080	2080	2077	2077

*N* (individuals) differs from *N* (observations) as multiple observations (remission ± relapse) per individual were included, according to each participant’s disease course

The odds ratios (OR, 95% CI, *p* value) are reported. The OR refers to the probability of having an AAV relapse (relative to remission)

Model 1 investigates the effect of *latitude*, adjusted for age at diagnosis, gender, AAV phenotype, ANCA serotype and treatment

Model 2 investigates the effect of *average winter (December to February) vitD-UVB (2004–2019)*, adjusted for age at diagnosis, gender, AAV phenotype, ANCA serotype and treatment

Model 3 investigates the effect of *average winter (December to February) CW-D-UVB (2004–2019)*, adjusted for age at diagnosis, gender, AAV phenotype, ANCA serotype and treatment

Model 4 investigates the effect of *average annual vitD-UVB*, adjusted for age at diagnosis, gender, AAV phenotype, ANCA serotype and treatment

Model 5 investigates the effect of *CW-D-UVB at symptom onset*, adjusted for age at diagnosis, gender, AAV phenotype, ANCA serotype and treatment

Model 6 investigates the effect of *average vitD-UVB over the preceding winter*, adjusted for age at diagnosis, gender, AAV phenotype, ANCA serotype and treatment

Model 7 investigates the effect of *average CW-D-UVB over the preceding winter*, adjusted for age at diagnosis, gender, AAV phenotype, ANCA serotype and treatment

*vitD-UVB* ambient UVB dose at wavelengths than induce vitD synthesis, *CW-D-UVB* cumulative-weighted UVB dose, *SD* standard deviation, *MPA* microscopic polyangiitis, *MPO* myeloperoxidase, *OR* odds ratio, *95% CI* 95% confidence interval

### Impact of latitude, measures of ambient vitD-UVB and cumulative-weighted UVB (CW-D-UVB) on AAV phenotype and serotype at diagnosis

We then investigated the effect of UVB exposure on AAV phenotype and serotype. Approximately half of the cases were diagnosed in winter/spring, with no demonstrable seasonal effect (Tables 1 and 2). UKIVAS and RKD participants reside at a relatively narrow latitudinal range, between 49.4–60.3° and 51.9–55.3°, respectively. Analyses of potential predictors of AAV phenotype and serotype are summarised in Tables 4 and Additional file 1: Table S4, respectively, and in Additional file 1: Figs. S7 and S8. We observed no significant associations between latitude (adjusted *p* = 0.39), average annual vitD-UVB (*p* = 0.65), average winter vitD-UVB (*p* = 0.46) or CW-D-UVB at symptom onset (*p* = 0.66) and being diagnosed with MPA or being MPO-ANCA positive. The findings were consistent in the sensitivity analysis when restricted to White participants (Additional file 1: Table S5). While non-significant, we found that vitD-UVB status at diagnosis (CW-D-UVB) was consistently below the annual average at the participants’ location (Additional file 1: Fig. S9).

**Table 4:** Uni- and multivariable logistic regression analysis of factors associated with AAV phenotype at diagnosis, in the combined UKIVAS and RKD cohort

		Not MPA	MPA	Unadjusted	Adjusted			
					Latitude	Average annual vitD-UVB	Average winter vitD-UVB	CW-D-UVB at symptom onset
					Model 1	Model 2	Model 3	Model 4
Age at diagnosis (years)	Mean (SD)	55.6 (15.0)	63.9 (13.7)	1.04 (1.04–1.05, ***p*** **< 0.001**)	1.04 (1.04–1.05, ***p*** **< 0.001**)	1.04 (1.04–1.05, ***p*** **< 0.001**)	1.04 (1.04–1.05, ***p*** **< 0.001**)	1.04 (1.04–1.05, ***p*** **< 0.001**)
Gender	Female	685 (63.1)	400 (36.9)	–	–	–	–	–
	Male	831 (67.7)	397 (32.3)	0.82 (0.69–0.97, ***p*** **= 0.022**)	0.83 (0.69–0.99, ***p*** **= 0.042**)	0.83 (0.69–0.99, ***p*** **= 0.041**)	0.83 (0.69–0.99, ***p*** **= 0.042**)	0.83 (0.69–0.99, ***p*** **= 0.039**)
Ethnicity	White	1415 (66.0)	728 (34.0)	–	–	–	–	–
	Asian	53 (58.9)	37 (41.1)	1.36 (0.88–2.08, *p* = 0.163)	2.01 (1.26–3.19, ***p*** **= 0.003**)	2.03 (1.28–3.22, ***p*** **= 0.003**)	2.03 (1.27–3.21, ***p*** **= 0.003**)	2.06 (1.29–3.25, ***p*** **= 0.002**)
	Black	13 (44.8)	16 (55.2)	2.39 (1.15–5.09, ***p*** **= 0.020**)	3.62 (1.67–8.02, ***p*** **= 0.001**)	3.67 (1.69–8.13, ***p*** **= 0.001**)	3.65 (1.68–8.07, ***p*** **= 0.001**)	3.69 (1.70–8.15, ***p*** **= 0.001**)
	Mixed	6 (66.7)	3 (33.3)	0.97 (0.20–3.69, *p* = 0.968)	1.27 (0.25–5.30, *p* = 0.754)	1.27 (0.25–5.34, *p* = 0.747)	1.27 (0.25–5.33, *p* = 0.750)	1.27 (0.25–5.33, *p* = 0.752)
	Others	29 (69.0)	13 (31.0)	0.87 (0.44–1.65, *p* = 0.683)	0.93 (0.45–1.82, *p* = 0.835)	0.94 (0.46–1.83, *p* = 0.857)	0.93 (0.45–1.82, *p* = 0.837)	0.95 (0.47–1.85, *p* = 0.886)
Latitude (degrees)	Mean (SD)	52.8 (1.6)	52.6 (1.5)	0.95 (0.90–1.01, *p* = 0.078)	0.98 (0.92–1.03, *p* = 0.392)	–	–	–
Average annual vitD-UVB (kJ/m^2^)	Mean (SD)	2.1 (0.2)	2.2 (0.2)	1.29 (0.88–1.91, *p* = 0.191)	–	1.10 (0.74–1.65, *p* = 0.645)	–	–
Average winter vitD-UVB (10 kJ/m^2^)	Mean (SD)	1.9 (0.4)	1.9 (0.3)	1.23 (0.96–1.58, *p* = 0.102)	–	–	1.10 (0.85–1.43, *p* = 0.458)	–
CW-D-UVB at symptom onset (J/m^2^)	Mean (SD)	0.1 (0.1)	0.1 (0.1)	0.67 (0.21–2.08, *p* = 0.487)	–	–	–	0.77 (0.23–2.51, *p* = 0.663)

The odds ratios (OR, 95% CI, *p* value) are reported. The OR refers to the probability of having MPA-AAV (ref: not MPA) at diagnosis

Model 1 investigates the effect of *latitude*, adjusted for age at diagnosis, gender and ethnicity (observations 2312, 1 missing age, AIC 2798.4)

Model 2 investigates the effect of *average annual vitD-UVB*, adjusted for age at diagnosis, gender and ethnicity (observations 2312, 1 missing age, AIC 2799)

Model 3 investigates the effect of *average winter (December to February) vitD-UVB (2004–2019)*, adjusted for age at diagnosis, gender and ethnicity (observations 2312, 1 missing age, AIC 2798.6)

Model 4 investigates the effect of *CW-D-UVB* at symptom onset, adjusted for age at diagnosis, gender and ethnicity (observations 2312, 1 missing age, AIC 2799)

*vitD-UVB* ambient UVB dose at wavelengths than induce vitD synthesis, *CW-D-UVB* cumulative-weighted UVB dose, *SD* standard deviation, *MPA* microscopic polyangiitis, *AAV* ANCA-associated vasculitis, *AIC* Akaike Information Criterion, *OR* odds ratio, *95% CI* 95% confidence interval

## Discussion

For the first time, we examined the relationships between latitude, measures of ambient vitD-UVB and UVB-predicted vitD status and AAV relapse risk. In keeping with other ADs, we demonstrated that low ambient UVB and, by extension, vitD deficiency are associated with *AAV relapse*, in a genetically homogeneous Irish cohort, after adjustment for key confounders. However, in this large registry-based study that included 2400 AAV patients from an ethnically and latitudinally restricted Irish and British cohort, we found no significant association between latitude, measures of ambient vitD-UVB or UVB-predicted vitD status (CW-D-UVB) and AAV *phenotype* or ANCA *serotype*.

While vitD deficiency has consistently been associated with increased disease activity in other ADs [21, 22], it has not previously been explored in depth in AAV. The risk of AAV relapse was positively associated with residential latitude and negatively with average vitD-UVB. The magnitude of the effect was greatest for average winter vitD-UVB, akin to the findings of Gatenby et al. [8]. This is biologically plausible as low winter exposure to vitD-UVB limits cutaneous vitD synthesis, particularly at more northerly latitudes [10]. We observed a similar negative correlation between average winter CW-D-UVB and relapse risk. For each 1 kJ/m^2^ increase in average winter CW-D-UVB, which equates to a 27.2 nmol/L rise in serum 25OHD [24], the odds of relapse decreased by 26%. Clinically, this is similar to moving from vitD ‘deficiency’ (< 25 nmol/L) to ‘sufficiency’ (> 50 nmol/L) [42].

We then explored narrower time intervals to pinpoint the at-risk window. While the average CW-D-UVB over the *preceding winter* (to the event) remained independently predictive of AAV relapse, the average vitD-UVB over the same period was not. The vitD-UVB nadir is in December, while CW-D-UVB (and serum 25OHD levels) continues to fall over ‘winter’, with a nadir 2 months later, so the effect of the latter may thus have been lost by restricting the analysis to winter. Importantly, we found no relationship between UVB-predicted vitD status (CW-D-UVB) at relapse onset and relapse risk. Together, these suggest that, an individual’s vitD status over a more prolonged period, particularly during winter, rather than their status peri-relapse, renders them susceptible to relapse and that multiple periods of low UVB and vitD deficiency might be contributing towards disease susceptibility. There was no significant interaction between AAV phenotype and any exploratory variable, suggesting there was no phenotype-specific effect, contrary to our hypothesis.

Drawing on incidence studies from 10 countries, Gatenby et al. [8] observed that GPA (but not MPA) incidence positively and negatively correlated with latitude (35.30° S to 70° N) and ambient UV radiation, respectively [8]. Consistent with this and other series [5–7], we observed a subtle (non-significant) increased odds of MPA (vs. GPA/EGPA) with decreasing latitude and increasing average vitD-UVB. It is possible that this *diagnosis* study component, using standard logistic regression, was underpowered due to the narrow latitudinal range in our cohort. However, contrary to suggestions that phenotype-specific UV effects are mediated by vitD effects on granuloma formation [8], we observed no relationship between UVB-predicted vitD status at symptom onset and AAV phenotype. As with AAV relapse, it may be an individual’s vitD status over a longer period, rather than immediately preceding disease onset, that is important. Critically, the outcome was MPA (vs. non-MPA) in this *diagnosis* study component. In our case-only cohorts, we could not directly interrogate whether UV radiation or UVB-predicted vitD status are implicated in *overall* AAV risk (i.e. AAV vs. non-AAV control). It is likely that vitD is important in de novo AAV onset but may not be responsible for defining the (non-) granulomatous phenotype.

The classification of AAV by ANCA serotype is increasingly preferred due to its enhanced predictive value in terms of relapse and renal survival [43, 44]. We found no significant association between latitude, average vitD-UVB or CW-D-UVB and ANCA serotype at diagnosis, although the direction of trends between latitude (negative)/vitD-UVB (positive) and MPO positivity was similar to that of MPA. This diverges from a multi-centre AAV glomerulonephritis registry-based cohort study that found anti-PR3 positivity was positively associated with latitude and negatively with UV radiation [9]. This study was comparable in size (*n* = 1408) to our cohort but covered a wider latitudinal range (Europe and the USA: 35.8° N to 69.6° N) and included a greater ethnic mix. In keeping with our findings, the significant association between ANCA serotype and latitude/UV radiation was lost when the analysis was restricted to centres in North and Central Europe, focusing on a more genetically homogeneous population. The observed variation may therefore be due to genetic or other differences between populations (± their interaction with UVB) as an alternative explanation for reported geographic patterns, for example, the distribution of the frequency of the HLA-DPB*0401 allele [45].

Our study is the first to investigate the effect of CW-D-UVB, a precise and well-validated vitD proxy, in AAV at an individual level, with high spatial and temporal resolution. This contrasts with the less refined vitD proxies used previously at a population level, such as latitude, sunshine hours or rainfall [8, 9]. The AAV relapse component of our study is the first of its kind to explore the role of ambient measures of vitD-UVB and UVB-predicted vitD on AAV *relapse*. For this, we exploited the benefits of the novel *n*-of-1 study design, primed for use in rare diseases [46], by providing increased power to detect associations by expanding control events. The case-crossover design allowed us to account for many unmeasured determinants of vitD status, such as skin type, lifestyle or SPF use, by comparing UVB measures in active and remission states within the same individual. We were also able to leverage detailed longitudinal immunosuppressive treatment data, enabling us to account for this as a time-varying confounder.

The limitations of our study must also be considered. Our predominantly White cohort originates from a relatively narrow Irish/UK latitudinal gradient. Consequently, restricted latitudinal and genetic variability may contribute to a type II error. Using ambient UVB dose rather than vitD has advantages and disadvantages. While CW-D-UVB as a vitD proxy has been validated in the UK biobank population of almost half a million participants [27], a community-dwelling Irish cohort > 60 years [26] and a RCT of inflammatory bowel disease [25], it has not been validated in AAV patients. Other factors beyond UVB contribute to vitD status: e.g. vitD supplementation might have been high in our cohort (due to its use in renal insufficiency and frequent over-the-counter use following strong public health campaigns), or disease-associated inflammation might have depleted vitD, potentially weakening the link between UVB and vitD status. We did not have data on vitD supplementation, sun exposure, time spent outside, clothing, SPF use, angle from the sun and many other factors—many of which would be impossible to obtain. However, we assumed each participant’s habits were consistent in the application of the multilevel model and the *n*-of-1 study design controls for such within-individual factors. Sporadic measurement of serum 25OHD concentration, the best marker of vitD status is of limited value, because it is strongly affected by season and disease activity [47] (reverse causation) and is not representative of longer-term trajectories. Therefore, a prospective cohort study examining the effect of vitD status on AAV *onset* is impossible (due to disease rarity) and a case-control study would be biassed by reverse causality (inflammation decreases vitD). Therefore, our study design using CW-D-UVB is valuable in the context of these limitations.

## Conclusion

In conclusion, we observed a strong inverse relationship between AAV *relapse* risk and individualised measures of vitD-UVB. A maximal effect was observed in winter. Prolonged low ambient UVB likely renders an individual vulnerable to a subsequent ‘hit’, which ultimately triggers disease activation. We found no evidence limiting this to granulomatous phenotypes. Our large study did not find any significant association between latitude, measures of vitD-UVB nor UVB-predicted vitamin D status and ANCA *phenotype* or *serotype*, albeit in an ethnically restricted cohort, across a narrow latitudinal window. It is plausible that prolonged vitD deficiency predisposes an individual to de novo AAV onset, but it does not appear to be important in the differentiation of AAV phenotype or serotype. Our observations have implications for the design of future vitD studies involving all relapsing-remitting diseases, not just AAV. We recommend a prospective cohort study to measure serial serum (or bloodspot) 25OHD at 3-monthly intervals to examine the trajectory of vitD in AAV *relapse* more accurately, focusing on average or winter vitD status. A RCT of vitD supplementation in AAV, targeting ‘sufficient’ 25OHD levels (>50nmol/L), with relapse as the primary outcome may then be warranted. vitD supplementation is generally well tolerated, safe and cheap to administer, but routine prescription should be avoided prior to definitive confirmation given the extra pill burden, unnecessary cost and potential for vitD toxicity with resultant hypercalcaemia.

## Supplementary Information

**Additional file 1: Fig. S1.** Flowchart of participant selection for the UKIVAS cohort (1). **Fig. S2.** Flowchart of participant selection for the RKD cohort (2). **Fig. S3.** Participant locations at diagnosis for i). UKIVAS and ii). RKD cohort. Shading represents the absolute number of participants recruited to the study in that region, at the time of their diagnosis. Tendency to cluster around vasculitis referral centres is observed. **Fig. S4.** Distribution of vitD-UVB (kJ/m2) (a and b) and CW-D-UVB (kJ/m2) (c and d) by month and season: CW-D-UVB peaks in late summer and nadirs in winter. **Fig. S5.** The relationship between latitude (degrees) and a). annual daily average vitD-UVB (kJ/m2, Pearson correlation coefficient (corr) -0.98, *p* <0.001), b). annual daily average CW-D-UVB (kJ/m2, corr -0.96, *p* <0.001). CW-D-UVB at diagnosis c). not adjusted for season (kJ/m2, corr -0.12, *p* <0.001), and d-g). stratified by season (corr winter -0.25, spring -0.15, summer -0.47, autumn -0.22, *p* <0.001 for all). **Fig. S6.** The relationship between latitude (degrees) and a). annual daily average vitD-UVB (kJ/m2, Pearson correlation coefficient (corr) -0.98, *p* <0.001), b). annual daily average CW-D-UVB (kJ/m2, corr -0.96, *p* <0.001). CW-D-UVB at diagnosis c). not adjusted for season (kJ/m2, corr -0.12, *p* <0.001), and d-g). stratified by season (corr winter -0.25, spring -0.15, summer -0.47, autumn -0.22, *p* <0.001 for all). **Fig. S7.** AAV diagnosis: a). Latitude (degrees), b). average annual vitD-UVB (kJ/m2), c). average winter vitD-UVB (kJ/m2) and d). CW-D-UVB (kJ/m2) stratified by AAV phenotype (GPA and EGPA (“Not MPA”) vs. MPA), at diagnosis. ANCA-associated vasculitis (AAV), Microscopic polyangiitis (MPA), Ambient UVB dose at wavelengths than induce vitD synthesis (vitD-UVB), Cumulative-weighted UVB dose (CW-D-UVB). **Fig. S8.** AAV diagnosis: Latitude (degrees), average annual vitD-UVB (kJ/m2), average winter vitD-UVB (kJ/m2) and CW-D-UVB (kJ/m2) stratified by AAV serotype (a-d: MPO vs. Not MPO), at diagnosis. ANCA-associated vasculitis (AAV), Myeloperoxidase (MPO), Cumulative-weighted UVB dose (CW-D-UVB). **Fig. S9.** Ratio of cumulative-weighted UVB at diagnosis relative to the average value at the participant’s location: a). cohort 1 & 2 and stratified by b). AAV phenotype and c). AAV serotype. ANCA-associated vasculitis (AAV), Microscopic polyangiitis (MPA), Myeloperoxidase (MPO), Cumulative-weighted UVB dose (CW-D-UVB), Not statistically significant (ns). If a true association between UVB-predicted vitD status (CW-D-UVB) and AAV phenotype/serotype at diagnosis exists, one would expect that the ratio of CW-D-UVB at diagnosis relative to the average CW-D-UVB at that location would be significantly <1 for non-MPA disease subtypes. A one-sided Wilcoxon rank sum test was used to evaluate if the ratio was significantly different from 1. While the median ratio trended below 1 in the pooled analysis and when stratified by AAV phenotype and serotype, this was not statistically different from 1. **Table S1.** Induction and maintenance treatment of RKD cohort (2). Intravenous (IV). **Table S2.** Multi-level model investigating the factors associated with AAV relapse risk, stratified by AAV phenotype (sensitivity analysis). N (individuals) differs from N (observations) as multiple observations (remission +/- relapse) per individual were included, according to each participant’s disease course. The OR refers to the probability of having an AAV relapse (relative to remission). Model 1 investigates the effect of average winter (Dec-Feb) vitD-UVB (2004-2019), adjusted for age at diagnosis, gender, AAV phenotype, ANCA serotype and treatment, restricted to MPA phenotype. Model 2 investigates the effect of average winter (Dec-Feb) vitD-UVB (2004-2019), adjusted for age at diagnosis, gender, AAV phenotype, ANCA serotype and treatment, restricted to Not MPA phenotype. Cumulative-weighted UVB dose (CW-D-UVB), Standard deviation (SD), Microscopic polyangiitis (MPA), Myeloperoxidase (MPO), Odds ratio (OR), 95% Confidence interval (95% CI). **Table S3.** Multi-level model investigating the association between CW-D-UVB and the risk of AAV relapse (with treatment breakdown). N (individuals) = 439, N (observations) = 2080 as multiple observations (remission +/- relapse) per individual were included according to each participant’s disease course. The OR refers to the probability of having an AAV relapse (relative to remission). Cumulative-weighted UVB dose (CW-D-UVB), Standard deviation (SD), Microscopic polyangiitis (MPA), Myeloperoxidase (MPO), Odds ratio (OR), 95% Confidence interval (95% CI), Azathioprine (AZA), Mycophenolate mofetil (MMF), Methotrexate (MTX). **Table S4.** Uni- and multivariable logistic regression analysis of factors associated with AAV serotype at diagnosis, in the combined UKIVAS and RKD cohort. Note: OR (95% CI, p). The OR refers to the probability of having MPO-positivity (ref: not MPO-ANCA) at diagnosis. Model 1 investigates the effect of latitude, adjusted for age at diagnosis, gender and ethnicity (observations 1260, 1053 missing ANCA serology, AIC 1626.9). Model 2 investigates the effect of average annual vitD-UVB, adjusted for age at diagnosis, gender and ethnicity (observations 1260, 1053 missing ANCA serology, AIC 1626.8). Model 3 investigates the effect of average winter (Dec-Feb) vitD-UVB (2004-2019), adjusted for age at diagnosis, gender and ethnicity (observations 1260, 1053 missing ANCA serology, AIC 1626.5). Model 4 investigates the effect of CW-D-UVB at symptom onset, adjusted for age at diagnosis, gender and ethnicity (observations 1260, 1053 missing ANCA serology, AIC 1627.3). Ambient UVB dose at wavelengths than induce vitD synthesis (vitD-UVB), Cumulative-weighted UVB dose (CW-D-UVB), Standard deviation (SD), Myeloperoxidase (MPO), Akaike information criterion (AIC), Odds ratio (OR), 95% Confidence interval (95% CI). **Table S5.** Uni- and multivariable logistic regression analysis of factors associated with AAV phenotype in the combined UKIVAS and RKD cohort, restricted to White participants (sensitivity analysis). Note: OR (95% CI, p). Observations = 2142 (1 missing age). The OR refers to the probability of having MPA-AAV (ref: not MPA) at diagnosis. Model 1 investigates the effect of latitude, adjusted for age at diagnosis and gender. Model 2 investigates the effect of average annual vitD-UVB, adjusted for age at diagnosis and gender. Model 3 investigates the effect of average winter (Dec-Feb) vitD-UVB (2004-2019), adjusted for age at diagnosis and gender. Model 4 investigates the effect of CW-D-UVB at symptom onset, adjusted for age at diagnosis and gender. Cumulative-weighted UVB dose (CW-D-UVB), Standard deviation (SD), Microscopic polyangiitis (MPA), ANCA-associated vasculitis (AAV), Akaike information criterion (AIC), Odds ratio (OR), 95% Confidence interval (95% CI). **Supplementary Methods**.

## Data Availability

Due to the rarity of ANCA-associated vasculitis, coupled with the identifiable nature of the data, raw data must remain confidential and cannot be freely shared. We would invite any potential research collaborations or data requests through the corresponding author on reasonable request.

## Abbreviations

ANCA: Anti-neutrophil cytoplasm antibody
AAV: ANCA-associated vasculitis
SVV: Small vessel vasculitis
AD: Autoimmune disease
MPA: Microscopic polyangiitis
GPA: Granulomatosis with polyangiitis
EGPA: Eosinophilic granulomatosis with polyangiitis
MPO: Myeloperoxidase
PR3: Proteinase-3
UV: Ultraviolet
vitD: Vitamin D
25OHD: 25-Hydroxyvitamin D
vitD-UVB: Ambient UVB dose at wavelengths that induce cutaneous vitD_3_ synthesis
CW-D-UVB: Cumulative-weighted ambient UVB dose
UKIVAS: United Kingdom and Ireland Vasculitis Rare Disease Group
RKD Registry and Biobank: Rare Kidney Disease Registry and Biobank
RDF: Resource Description Framework
TEMIS: Tropospheric Emission Monitoring Internet Service
ORs: Odds ratios
95% CI: 95% confidence interval
SD: Standard deviation
IQR: Inter-quartile range
AIC: Akaike Information Criterion
ELISA: Enzyme-linked immunoassay
ns: Not statistically significant
RCT: Randomised controlled trial
