# Supplementary material for: The association between ambient UVB dose and ANCA-associated vasculitis relapse and onset
Source: Arthritis Res Ther. 2022 Jun 18;24:147. doi: 10.1186/s13075-022-02834-6 (PMC9206351; doi:10.1186/s13075-022-02834-6)
Supplement: Supplementary file 1 — Additional file 1: Fig. S1. Flowchart of participant selection for the UKIVAS cohort (1). Fig. S2. Flowchart of participant selection for the RKD cohort (2). Fig. S3. Participant locations at diagnosis for i). UKIVAS and ii). RKD cohort. Shading represents the absolute number of participants recruited to the study in that region, at the time of their diagnosis. Tendency to cluster around vasculitis referral centres is observed. Fig. S4. Distribution of vitD-UVB (kJ/m2) (a and b) and CW-D-UVB (kJ/m2) (c and d) by month and season: CW-D-UVB peaks in late summer and nadirs in winter. Fig. S5. The relationship between latitude (degrees) and a). annual daily average vitD-UVB (kJ/m2, Pearson correlation coefficient (corr) -0.98, p <0.001), b). annual daily average CW-D-UVB (kJ/m2, corr -0.96, p <0.001). CW-D-UVB at diagnosis c). not adjusted for season (kJ/m2, corr -0.12, p <0.001), and d-g). stratified by season (corr winter -0.25, spring -0.15, summer -0.47, autumn -0.22, p <0.001 for all). Fig. S6. The relationship between latitude (degrees) and a). annual daily average vitD-UVB (kJ/m2, Pearson correlation coefficient (corr) -0.98, p <0.001), b). annual daily average CW-D-UVB (kJ/m2, corr -0.96, p <0.001). CW-D-UVB at diagnosis c). not adjusted for season (kJ/m2, corr -0.12, p <0.001), and d-g). stratified by season (corr winter -0.25, spring -0.15, summer -0.47, autumn -0.22, p <0.001 for all). Fig. S7. AAV diagnosis: a). Latitude (degrees), b). average annual vitD-UVB (kJ/m2), c). average winter vitD-UVB (kJ/m2) and d). CW-D-UVB (kJ/m2) stratified by AAV phenotype (GPA and EGPA (“Not MPA”) vs. MPA), at diagnosis. ANCA-associated vasculitis (AAV), Microscopic polyangiitis (MPA), Ambient UVB dose at wavelengths than induce vitD synthesis (vitD-UVB), Cumulative-weighted UVB dose (CW-D-UVB). Fig. S8. AAV diagnosis: Latitude (degrees), average annual vitD-UVB (kJ/m2), average winter vitD-UVB (kJ/m2) and CW-D-UVB (kJ/m2) stratified by AAV serotype (a-d: MPO vs. Not MPO), a [file 13075_2022_2834_MOESM1_ESM.docx]

**Supplementary Materials**

**Table of Contents**

Supplementary Figures 3

Supplementary Figure 1: Flowchart of participant selection for the UKIVAS cohort (1) 3

Supplementary Figure 2: Flowchart of participant selection for the RKD cohort (2) 4

Supplementary Figure 3: Participant locations at diagnosis for i). UKIVAS and ii). RKD cohort 5

Supplementary Figure 4: Distribution of vitD-UVB (kJ/m2) (a and b) and CW-D-UVB (kJ/m2) (c and d) by month and season: CW-D-UVB peaks in late summer and nadirs in winter. 6

Supplementary Figure 5: The relationship between latitude (degrees) and a). annual daily average vitD-UVB (kJ/m2, Pearson correlation coefficient (corr) -0.98, *p* <0.001), b). annual daily average CW-D-UVB (kJ/m2, corr -0.96, *p* <0.001). CW-D-UVB at diagnosis c). not adjusted for season (kJ/m2, corr -0.12, *p* <0.001), and d-g). stratified by season (corr winter -0.25, spring -0.15, summer -0.47, autumn -0.22, *p* <0.001 for all). 7

Supplementary Figure 6: *AAV relapse:* a). CW-D-UVB at symptom onset (kJ/m2), b). average preceding winter vitD-UVB (kJ/m2) and c). average preceding winter CW-D-UVB (kJ/m2) stratified by disease activity (Active vs. Remission) in the entire cohort 2. 8

Supplementary Figure 7: *AAV diagnosis:* a). Latitude (degrees), b). average annual vitD-UVB (kJ/m2), c). average winter vitD-UVB (kJ/m2) and d). CW-D-UVB (kJ/m2) stratified by AAV phenotype (GPA and EGPA (“Not MPA”) vs. MPA), *at diagnosis*. 9

Supplementary Figure 8: *AAV diagnosis:* Latitude (degrees), average annual vitD-UVB (kJ/m2), average winter vitD-UVB (kJ/m2) and CW-D-UVB (kJ/m2) stratified by AAV serotype (a-d: MPO vs. Not MPO), *at diagnosis*. 10

ANCA-associated vasculitis (AAV), Myeloperoxidase (MPO), Cumulative-weighted UVB dose (CW-D-UVB) 10

Supplementary Figure 9: Ratio of cumulative-weighted UVB at diagnosis relative to the average value at the participant’s location: a). cohort 1 & 2 and stratified by b). AAV phenotype and c). AAV serotype 11

Supplementary Tables 12

Supplementary Table 1: Induction and maintenance treatment of RKD cohort (2) 12

Supplementary Table 2: Multi-level model investigating the factors associated with AAV relapse risk, stratified by AAV phenotype (sensitivity analysis) 13

Supplementary Table 3: Multi-level model investigating the association between CW-D-UVB and the risk of AAV relapse (with treatment breakdown) 14

Supplementary Table 5*:*Uni- and multivariable logistic regression analysis of factors associated with AAV phenotype in the combined UKIVAS and RKD cohort, restricted to White participants (sensitivity analysis) 17

Supplementary Methods 18

Ultraviolet B (UVB) data resource: TEMIS 18

Cumulative-weighted UVB dose (CW-D-UVB) 18

Study design: AAV relapse 19

Study Design: AAV diagnosis: 77-day prodrome between date of symptom onset and diagnosis 19

Patient and public involvement 19

Statistical analysis 19

Additional References 20

# Supplementary Figures

## Supplementary Figure 1: Flowchart of participant selection for the UKIVAS cohort (1)

## Supplementary Figure 2: Flowchart of participant selection for the RKD cohort (2)

## Supplementary Figure 3: Participant locations at diagnosis for i). UKIVAS and ii). RKD cohort

Shading represents the absolute number of participants recruited to the study in that region, at the time of their diagnosis.
Tendency to cluster around vasculitis referral centres is observed.

## Supplementary Figure 4: Distribution of vitD-UVB (kJ/m2) (a and b) and CW-D-UVB (kJ/m2) (c and d) by month and season: CW-D-UVB peaks in late summer and nadirs in winter.

## Supplementary Figure 5: The relationship between latitude (degrees) and a). annual daily average vitD-UVB (kJ/m2, Pearson correlation coefficient (corr) -0.98, *p* <0.001), b). annual daily average CW-D-UVB (kJ/m2, corr -0.96, *p* <0.001). CW-D-UVB at diagnosis c). not adjusted for season (kJ/m2, corr -0.12, *p* <0.001), and d-g). stratified by season (corr winter -0.25, spring -0.15, summer -0.47, autumn -0.22, *p* <0.001 for all).

##

## Supplementary Figure 6: *AAV relapse:* a). CW-D-UVB at symptom onset (kJ/m2), b). average preceding winter vitD-UVB (kJ/m2) and c). average preceding winter CW-D-UVB (kJ/m2) stratified by disease activity (Active vs. Remission) in the entire cohort 2.

Ambient UVB dose at wavelengths than induce vitD synthesis (vitD-UVB), Cumulative-weighted UVB dose (CW-D-UVB)

## Supplementary Figure 7: *AAV diagnosis:* a). Latitude (degrees), b). average annual vitD-UVB (kJ/m2), c). average winter vitD-UVB (kJ/m2) and d). CW-D-UVB (kJ/m2) stratified by **AAV phenotype** (GPA and EGPA (“Not MPA”) vs. MPA), *at diagnosis*.

ANCA-associated vasculitis (AAV), Microscopic polyangiitis (MPA), Ambient UVB dose at wavelengths than induce vitD synthesis (vitD-UVB), Cumulative-weighted UVB dose (CW-D-UVB)

## Supplementary Figure 8: *AAV diagnosis:* Latitude (degrees), average annual vitD-UVB (kJ/m2)**,** average winter vitD-UVB (kJ/m2) and CW-D-UVB (kJ/m2) stratified by **AAV serotype** (a-d: MPO vs. Not MPO), *at diagnosis*.

## ANCA-associated vasculitis (AAV), Myeloperoxidase (MPO), Cumulative-weighted UVB dose (CW-D-UVB)

## Supplementary Figure 9: Ratio of cumulative-weighted UVB at diagnosis relative to the average value at the participant’s location: a). cohort 1 & 2 and stratified by b). AAV phenotype and c). AAV serotype

ANCA-associated vasculitis (AAV), Microscopic polyangiitis (MPA), Myeloperoxidase (MPO), Cumulative-weighted UVB dose (CW-D-UVB), Not statistically significant (ns).
If a true association between UVB-predicted vitD status (CW-D-UVB) and AAV phenotype/serotype at diagnosis exists, one would expect that the ratio of CW-D-UVB at diagnosis relative to the average CW-D-UVB at that location would be significantly <1 for non-MPA disease subtypes. A one-sided Wilcoxon rank sum test was used to evaluate if the ratio was significantly different from 1. While the median ratio trended below 1 in the pooled analysis and when stratified by AAV phenotype and serotype, this was not statistically different from 1.

# Supplementary Tables

## Supplementary Table 1: Induction and maintenance treatment of RKD cohort (2)

| **Characteristics** | **Total** | **GPA** | **MPA** | **EGPA** | ***p*** |
| --- | --- | --- | --- | --- | --- |
| *n (%)* | 439 | 196 (44.6) | 220 (50.1) | 23 (5.2) |  |
| *Induction treatment (%)* |  |  |  |  |  |
| Oral corticosteroids | 400 (91.1) | 178 (90.8) | 200 (90.9) | 22 (95.7) | 0.922 |
| IV corticosteroids | 233 (53.1) | 100 (51.0) | 124 (56.4) | 9 (39.1) | 0.214 |
| Oral cyclophosphamide | 175 (39.9) | 92 (46.9) | 74 (33.6) | 9 (39.1) | 0.022 |
| IV cyclophosphamide | 138 (31.4) | 57 (29.1) | 78 (35.5) | 3 (13.0) | 0.055 |
| Rituximab | 92 (21.0) | 34 (17.3) | 56 (25.5) | 2 (8.7) | 0.046 |
| Methotrexate | 17 (3.9) | 5 (2.6) | 9 (4.1) | 3 (13.0) | 0.062 |
| Other | 48 (10.9) | 26 (13.3) | 15 (6.8) | 7 (30.4) | 0.096 |
| Plasma exchange | 90 (20.5) | 41 (20.9) | 48 (21.8) | 1 (4.3) | 0.13 |
| *Maintenance treatment (%)* |  |  |  |  |  |
| Oral corticosteroids | 365 (83.1) | 165 (84.2) | 178 (80.9) | 22 (95.7) | 0.184 |
| Azathioprine | 243 (55.4) | 114 (58.2) | 117 (53.2) | 12 (52.2) | 0.566 |
| Rituximab | 94 (21.4) | 55 (28.1) | 36 (16.4) | 3 (13.0) | 0.01 |
| Mycophenolate Mofetil | 89 (20.3) | 36 (18.4) | 44 (20.0) | 9 (39.1) | 0.064 |
| Methotrexate | 49 (11.2) | 37 (18.9) | 10 (4.5) | 2 (8.7) | <0.001 |
| Other | 28 (6.4) | 16 (8.2) | 9 (4.1) | 3 (13.0) | 0.052 |

Intravenous (IV)

## Supplementary Table 2: Multi-level model investigating the factors associated with AAV relapse risk, **stratified by AAV phenotype (sensitivity analysis)**

|  | **Model 1 (MPA)** | **Model 2 (Not MPA)** |
| --- | --- | --- |
| **Random effects (Variance (SD))** |  |  |
| Patient ID | 0.98 (0.99) | 0.38 (0.62) |
| **Fixed effects (OR (95% CI, *p*))** |  |  |
| Average winter vitD-UVB (kJ/m2) | 0.59 (0.36-0.98, **0.04)** | 0.77 (0.60-0.98, **0.03**) |
| Age at diagnosis (years) | 0.68 (0.46-1.01, 0.06) | 0.74 (0.58-0.95, **0.02**) |
| Gender (male) | 0.63 (0.29-1.36, 0.24) | 1.12 (0.69-1.83, 0.64) |
| Not MPO-ANCA (*Ref: MPO-ANCA*) | 1.56 (0.67-3.65, 0.30) | 0.78 (0.40-1.52, 0.47) |
| Off treatment (*Ref: On treatment*) | 2.91 (1.26-6.71, **0.01)** | 2.64 (1.56-4.46, **<0.001)** |
| Number of individuals | 220 | 219 |
| Number of observations | 990 | 1090 |

N (individuals) differs from N (observations) as multiple observations (remission +/- relapse) per individual were included, according to each participant’s disease course. The OR refers to the probability of having an AAV relapse (relative to remission).

Model 1 investigates the effect of **average winter (Dec-Feb) vitD-UVB (2004-2019),** adjusted for age at diagnosis, gender, AAV phenotype, ANCA serotype and treatment, **restricted to *MPA phenotype***.

Model 2 investigates the effect of **average winter (Dec-Feb) vitD-UVB (2004-2019),** adjusted for age at diagnosis, gender, AAV phenotype, ANCA serotype and treatment, **restricted to *Not MPA phenotype.***

Cumulative-weighted UVB dose (CW-D-UVB), Standard deviation (SD), Microscopic polyangiitis (MPA), Myeloperoxidase (MPO), Odds ratio (OR), 95% Confidence interval (95% CI)

## Supplementary Table 3: Multi-level model investigating the association between CW-D-UVB and the risk of AAV relapse (with treatment breakdown)

| **Random effects:** | **Variance (SD)** |
| --- | --- |
| Patient ID | 0.75 (0.86 |
| **Fixed effects:** | **OR (95% CI, p)** |
| CW-D-UVB (kJ/m2) | 1.20 (0.87 - 1.66, 0.28) |
| Not MPA (Ref: MPA) | 1.69 (0.95 - 2.98, **0.07**) |
| Age at diagnosis (years) | 0.72 (0.58 - 0.90, **0.003**) |
| Gender (male) | 0.89 (0.58 - 1.37, 0.60) |
| Not MPO-ANCA (Ref: MPO-ANCA) | 1.10 (0.63 - 1.92, 0.73) |
| *Treatment: (Ref: AZA/MMF/MTX/Other)* | |
| Rituximab | 0.41 (0.16 - 1.03, 0.06) |
| Cyclophosphamide | 0.58 (1.25 - 2.66, 0.50) |
| Glucocorticoid monotherapy | 1.85 (1.02 - 3.35**, 0.04**) |
| Off treatment | 2.69 (1.65 - 4.40, **<0.001**) |
| CW-D-UVB: Not MPA (Ref: MPA) | 0.83 (0.56 - 1.24, 0.36) |

N (individuals) = 439, N (observations) = 2080 as multiple observations (remission +/- relapse) per individual were included according to each participant’s disease course. The OR refers to the probability of having an AAV relapse (relative to remission). Cumulative-weighted UVB dose (CW-D-UVB), Standard deviation (SD), Microscopic polyangiitis (MPA), Myeloperoxidase (MPO), Odds ratio (OR), 95% Confidence interval (95% CI), Azathioprine (AZA), Mycophenolate mofetil (MMF), Methotrexate (MTX)

Supplementary Table 4:  Uni- and multivariable logistic regression analysis of factors associated with AAV serotype at diagnosis, in the combined UKIVAS and RKD cohort

|  |  | Not MPO | MPO | Unadjusted |  | Adjusted |  |  |
| --- | --- | --- | --- | --- | --- | --- | --- | --- |
|  |  |  |  |  | **Latitude** | **Average annual vitD-UVB** | **Average winter vitD-UVB** | **CW-D-UVB at symptom onset** |
|  |  |  |  |  | **Model 1** | **Model 2** | **Model 3** | **Model 4** |
| Age at diagnosis (yrs) | Mean (SD) | 55.6 (15.8) | 63.4 (12.9) | 1.04 (1.03-1.05, **p<0.001**) | 1.04 (1.03-1.05, **p<0.001**) | 1.04 (1.03-1.05, **p<0.001**) | 1.04 (1.03-1.05, **p<0.001**) | 1.04 (1.03-1.05, **p<0.001**) |
| Gender | Female | 329 (56.9) | 249 (43.1) | - | - | - | - | - |
|  | Male | 417 (61.1) | 265 (38.9) | 0.84 (0.67-1.05, p=0.129) | 0.84 (0.67-1.06, p=0.149) | 0.84 (0.67-1.06, p=0.150) | 0.84 (0.67-1.06, p=0.150) | 0.84 (0.66-1.06, p=0.141) |
| Ethnicity | White | 690 (59.4) | 472 (40.6) | - | - | - | - | - |
|  | Asian | 30 (60.0) | 20 (40.0) | 0.97 (0.54-1.73, p=0.930) | 1.41 (0.75-2.59, p=0.278) | 1.41 (0.76-2.60, p=0.271) | 1.44 (0.75-2.58, p=0.279) | 1.44 (0.77-2.64, p=0.245) |
|  | Black | 9 (52.9) | 8 (47.1) | 1.30 (0.48-3.42, p=0.593) | 1.71 (0.61-4.69, p=0.296) | 1.71 (0.61-4.70, p=0.293) | 1.70 (0.61-4.67, p=0.297) | 1.75 (0.63-4.80, p=0.271) |
|  | Mixed | 2 (40.0) | 3 (60.0) | 2.19 (0.36-16.70, p=0.391) | 2.23 (0.34-17.95, p=0.401) | 2.23 (0.34-17.97, p-0.401) | 2.27 (0.34-17.91, p=0.402) | 2.27 (0.35-18.33, p=0.391) |
|  | Other | 15 (57.7) | 11 (42.3) | 1.07 (0.48-2.34, p=0.862) | 1.15 (0.50-2.61, p=0.730) | 1.15 (0.50-2.61, p=0.731) | 1.14 (0.49-2.58, p=0.752) | 1.18 (0.51-2.66, p=0.685) |
| Latitude (degrees) | Mean (SD) | 52.8 (1.6) | 52.7 (1.5) | 0.95 (0.88-1.02, p=0.149) | 0.97 (0.90-1.05, p=0.501) | - | - | - |
| Average annual vitD-UVB (kJ/m2) | Mean (SD) | 2.1 (0.2) | 2.1 (0.2) | 1.47 (0.88-2.48, p=0.143) | - | 1.22 (0.71-2.10, p=0.477) | - | - |
| Average winter vitD-UVB  (10 kJ/m2) | Mean (SD) | 1.8 (0.3) | 1.9 (0.3) | 1.33 (0.96-1.87, p=0.092) | - | - | 1.18 (0.83-1.67, p=0.362) | - |
| CW-D-UVB at symptom onset (J/m2) | Mean (SD) | 0.1 (0.1) | 0.1 (0.1) | 0.82 (0.18-3.67, p=0.791) | - | - | - | 0.86 (0.18-4.12, p=0.855) |

Note: OR (95% CI, p)

The OR refers to the probability of having MPO-positivity (ref: not MPO-ANCA) at diagnosis.

Model 1 investigates the effect of **latitude**, adjusted for age at diagnosis, gender and ethnicity (observations 1260, 1053 missing ANCA serology, AIC 1626.9).

Model 2 investigates the effect of **average annual vitD-UVB,** adjusted for age at diagnosis, gender and ethnicity (observations 1260, 1053 missing ANCA serology, AIC 1626.8).

Model 3 investigates the effect of **average winter (Dec-Feb) vitD-UVB (2004-2019),** adjusted for age at diagnosis, gender and ethnicity (observations 1260, 1053 missing ANCA serology, AIC 1626.5).

Model 4 investigates the effect of **CW-D-UVB** at symptom onset, adjusted for age at diagnosis, gender and ethnicity (observations 1260, 1053 missing ANCA serology, AIC 1627.3).

## Ambient UVB dose at wavelengths than induce vitD synthesis (vitD-UVB), Cumulative-weighted UVB dose (CW-D-UVB), Standard deviation (SD), Myeloperoxidase (MPO), Akaike information criterion (AIC), Odds ratio (OR), 95% Confidence interval (95% CI)

## Supplementary Table 5*:*Uni- and multivariable logistic regression analysis of factors associated with AAV phenotype in the combined UKIVAS and RKD cohort, **restricted to White participants (sensitivity analysis)**

|  |  | Not MPA  (ref) | MPA | Unadjusted |  | Adjusted | |  |  |
| --- | --- | --- | --- | --- | --- | --- | --- | --- | --- |
|  |  |  |  |  | **Latitude** | **Average annual vitD-UVB** | **Average winter vitD-UVB** | | **CW-D-UVB at symptom onset** |
|  |  |  |  |  | **Model 1** | **Model 2** | **Model 3** | | **Model 4** |
| Age at diagnosis (years) | Mean (SD) | 56.2 (14.8) | 64.4 (13.3) | 1.04 (1.04-1.05, p<0.001) | 1.04 (1.04-1.05, p<0.001) | 1.04 (1.04-1.05, p<0.001) | 1.04 (1.04-1.05, p<0.001) | | 1.04 (1.04-1.05, p<0.001) |
| Gender | Female | 639 (64.4) | 354 (35.6) | - | - |  | - | | - |
|  | Male | 776 (67.5) | 374 (32.5) | 0.87 (0.73-1.04, p=0.127) | 0.90 (0.74-1.08, p=0.246) | 0.90 (0.74-1.08, p=0.243) | 0.90 (0.74-1.08, p=0.246) | | 0.89 (0.74-1.08, p=0.241) |
| Latitude (degrees) | Mean (SD) | 52.8 (1.6) | 52.7 (1.5) | 0.97 (0.92-1.03, p=0.297) | 0.98 (0.93-1.04, p=0.587) |  | - | | - |
| Average annual vitD-UVB (kJ/m2) | Mean (SD) | 2.1 (0.2) | 2.1 (0.2) | 1.16 (0.78-1.72, p0.470) |  | 1.05 (0.70-1.59, p=0.801) |  | |  |
| Average winter vitD-UVB (10kJ/m2) | Mean (SD) | 1.8 (0.4) | 1.9 (0.3) | 1.14 (0.89-1.47, p=0.312) | - |  | 1.07 (0.82-1.39, p=0.624) | | - |
| CW-D-UVB (J/m2) | Mean (SD) | 0.1 (0.1) | 0.1 (0.1) | 0.52 (0.16-1.70, p=0.279) | - |  | - | | 0.61 (0.18-2.10, p=0.438) |
| AIC |  |  |  |  | 2589.7 | 2590 | 2590 | | 2591.4 |

Note: OR (95% CI, p). Observations = 2142 (1 missing age). The OR refers to the probability of having MPA-AAV (ref: not MPA) at diagnosis.

Model 1 investigates the effect of **latitude**, adjusted for age at diagnosis and gender.

Model 2 investigates the effect of **average annual vitD-UVB,** adjusted for age at diagnosis and gender.

Model 3 investigates the effect of **average winter (Dec-Feb) vitD-UVB (2004-2019),** adjusted for age at diagnosis and gender.

Model 4 investigates the effect of **CW-D-UVB** at symptom onset, adjusted for age at diagnosis and gender.

Cumulative-weighted UVB dose (CW-D-UVB), Standard deviation (SD), Microscopic polyangiitis (MPA), ANCA-associated vasculitis (AAV), Akaike information criterion (AIC), Odds ratio (OR), 95% Confidence interval (95% CI)

#

# Supplementary Methods

## Ultraviolet B (UVB) data resource: TEMIS

UV data, obtained from TEMIS, is computed daily using satellite data relating to the intensity of solar radiation, global ozone columns and weather data. The UV dose data is provided on a rectangular longitude x latitude grid of 0.25 x 0.25 degrees. The cloud data is compiled from geostationary Meteosat Second Generation (MSG) observations. On days when cloud data is missing, the cloud-modified UV dose is replaced by the climatological value (average over 2004-2019, requiring >10 year of data being available). If that climatological value is not available, then 0.7 times the clear-sky UV dose of the day in question is used as a first order estimate. UVB is a very narrow band within the total UV radiation spectrum, with the action spectrum for vitD synthesis between 280-315 nm, and peak conversion between at 295-298 nm[1]. The daily cloud-adjusted ambient UV dose was then computed for vitD-effective wavelengths resulting in the daily ambient dose of UV radiation (reaching the earth’s surface) that is capable of inducing vitD synthesis in the skin (vitD-UVB). UV data was unavailable prior to 1^st^ January 2004. Given the algorithm cumulates data for a 135-day period, event dates prior to 15^th^ May 2004 were excluded from the analysis. Seasons were defined as: winter (Dec-Feb), spring (Mar- May), summer (Jun-Aug) and autumn (Sep-Nov).

## Cumulative-weighted UVB dose (CW-D-UVB)

To generate UVB-predicted vitD status a specific cumulative-weighted UVB dose (CW-D-UVB) was calculated for each participant, determined by high-resolution location and date based on an algorithm proposed by Kelly et al[2] and later validated in an older Irish cohort[3], a vitD Randomised Controlled Trial (RCT) setting[4] and UK Biobank[5]. Specifically, this function accumulates the cloud-modified UVB dose at wavelengths specific for vitD synthesis (vitD-UVB) over a period of 135 days prior to a given date, with a decay function assuming a half-life of vitD of 35 days. The decay function (**Figure 1**) mimics the physiological accumulation (summer months) and depletion (winter) of vitD: body stores increase at times of UVB abundance (i.e. summer) and diminish during periods of low UVB radiation. The CW-D-UVB dose estimate is unique for each participant and for each disease state as the estimates are determined by time and location. The time and location precision of these estimates incorporates the substantial geotemporal variations (seasonal and latitudinal) observed in vitD-UVB, despite the small latitudinal differences across Ireland (51°30’N - 55°24’N)[2, 3] and the UK (49°42’N - 60°31’N). CW-D-UVB at symptom onset and at discrete times of relapse and remission were calculated for each participant. The average (2004-2019) and preceding winter CW-D-UVB were also derived as for vitD-UVB.

## Study design: AAV relapse

The design of our study is dependent on an accurate assessment of the date of relapse onset. To estimate the delay between relapse symptom onset and diagnosis, a patient focus group was established through Vasculitis Ireland Awareness^[[1]](#footnote-2)^. Based on these discussions, an interval of 30 days was used in our model development (Figure 2).

The 135-day period after relapse was selected to avoid the influence of residual disease activity on CW-D-UVB exposure estimate (which is based on the preceding 135 days). The control date was defined as a randomly selected date within a period of definite remission, ensuring no relapse event occurred in the 135 days prior or 90 days after this date. 90 days post was chosen as a washout period, as we envisaged any potential relapse would have been identified at the standard interval 3-monthly visits.

## Study Design: AAV diagnosis: 77-day prodrome between date of symptom onset and diagnosis

Of 600 definite AAV cases in the RKD registry, date of both symptom onset and diagnosis were available for 370 participants. The median prodrome was 77 days (IQR 31-216). The prodrome was longest for those whom symptoms began in winter (104 days, IQR 42-290) and shortest for those commencing in spring (57 days, IQR 28-188). Prodromal duration also varied by AAV phenotype: EGPA 188 days (IQR 75.5-1200), GPA 92 days (37.2-253) and MPA 62 days (IQR 26-181).

## Patient and public involvement

Patient involvement was primarily through the national patient group, ‘Vasculitis Ireland Awareness’, through focus groups and ‘question and answer’ sessions at their national annual meeting. Patients were instrumental in prioritising the research question which led to this study. AAV relapse is a recognised target of research for the wider vasculitis community also. Patients were involved in the design and conduct of this research. Julie Power, a patient representative, joined the study steering committee. We developed a study newsletter in conjunction with VIA (hosted on their website: <https://vasculitis-ia.org/>) to inform participants and the wider vasculitis community about study updates. We will disseminate study findings through this website, social media channels and at a patient session at the International Vasculitis and ANCA Workshop (April 2022).

## Statistical analysis

**AAV relapse power calculation.** This was performed using simulation which characterised a remitting relapsing pattern dependent on a seasonal CW-D-UVB component mimicking the n-of-1 sampling design used. Similar approaches for the vitD trial design are described in[6]. With *n*=439 we have an 85% power to detect an effect when a 10 unit increase CW-D-UVB results in an odds ratio of relapse of 0.55.

Of note, continuous variables were scaled (*scale* function in R: subtract mean and divide by standard deviation for each value) prior to inclusion in the multi-level models.

**AAV diagnosis.**

i). *CW-D-UVB at diagnosis was normalised to the average annual CW-D-UVB at that location*, thereby accounting for latitudinal differences in CW-D-UVB . Both the ratio and delta were calculated, and one-sided Wilcoxon rank sum tests were performed to determine if the median was significantly different from 1 and 0, respectively.

## Additional References

1. Bouillon R EJ, GarabedianM, Holick MF, Kleinschmidt J, Suda T,, Terenetskaya I WA: **Action spectrum for the production of previtamin D3 in human skin.** In*.* Vienna (Austria): International Commission on Illumination; 2006.

2. Kelly D, Theodoratou E, Farrington SM, Fraser R, Campbell H, Dunlop MG, Zgaga L: **The contributions of adjusted ambient ultraviolet B radiation at place of residence and other determinants to serum 25-hydroxyvitamin D concentrations**. *British Journal of Dermatology* 2016, **174**(5):1068-1078.

3. O'Sullivan F, Laird E, Kelly D, van Geffen J, van Weele M, McNulty H, Hoey L, Healy M, McCarroll K, Cunningham C *et al*: **Ambient UVB Dose and Sun Enjoyment Are Important Predictors of Vitamin D Status in an Older Population**. *The Journal of Nutrition* 2017, **147**(5):858-868.

4. O'Sullivan F, Raftery T, van Weele M, van Geffen J, McNamara D, O'Morain C, Mahmud N, Kelly D, Healy M, O'Sullivan M *et al*: **Sunshine is an Important Determinant of Vitamin D Status Even Among High-dose Supplement Users: Secondary Analysis of a Randomized Controlled Trial in Crohn's Disease Patients**. *Photochemistry and Photobiology* 2019, **95**(4):1060-1067.

5. Li X, Geffen Jv, Weele Mv, Zhang X, He Y, Meng X, Timofeeva M, Campbell H, Dunlop M, Zgaga L *et al*: **Genetically-predicted vitamin D status, ambient UVB during the pandemic and COVID-19 risk in UK Biobank: Mendelian Randomisation study**. *medRxiv* 2020:2020.2008.2018.20177691.

6. Wyse J, Mangan R, Zgaga L: **Power determination in vitamin D randomised control trials and characterising factors affecting it through a novel simulation-based tool**. *Scientific Reports* 2021, **11**(1):10804.

1. https://vasculitis-ia.org/ [↑](#footnote-ref-2)
